# Supplementary material for: Extracting and visualizing hidden activations and computational graphs of PyTorch models with TorchLens
Source: Sci Rep. 2023 Sep 1;13:14375. doi: 10.1038/s41598-023-40807-0 (PMC10474256; doi:10.1038/s41598-023-40807-0)
Supplement: Supplementary file 1 — Supplementary Table 1. [file 41598_2023_40807_MOESM1_ESM.pdf]

Supplementary Information

Supplementary Table 1: Model and layer metadata provided by *TorchLens*. Entries in the left column are attributes of the ModelHistory object returned by *TorchLens*; entries in the right are attributes of the log entry for each individual operation that can be fetched from ModelHistory.

Model Metadata	Operation Metadata
model_name: name of model class	*layer_label*: the label for the layer (e.g., conv2d_3_5)
random_seed_used: random seed used during forward pass	*layer_label_w_pass*: the label for the layer along with the pass number (e.g., conv2d_3_5:4 for the 4th pass)
model_is_recurrent: whether the model contains recurrent layers	*operation_num*: how many tensor operations have been performed up to that layer (e.g., 6 for the sixth operation)
model_max_recurrent_loops: the maximum number of recurrent passes for any layer in the model	*layer_type*: The type of operation performed (e.g., conv2d)
model_is_branching: whether the model contains branching (i.e., a layer has more than one child layer)	*layer_type_num*: how many layers of that type have appeared in the model up to that layer (e.g., 2 if the layer is the second convolutional layer)
model_has_conditional_branching: whether the model contains if-then branching	*layer_total_num*: how many total layers have appeared in the model up to that layer (e.g., 4 for the fourth layer overall)
layer_list: list of layer names	*pass_num*: the pass number of the layer for that operation (e.g., 3 if the operation is the third pass of a layer)
input_layers: list of model input layers	*layer_passes_total*: total number of passes for the layer
output_layers: list of model output layers	*lookup_keys*: list of valid keys for indexing the operation (e.g., the layer name, the module address, the layer's ordinal position in the model, etc.)
buffer_layers: list of model buffer layers	*tensor_contents*: the saved tensor for that operation
internally_initialized_layers: list of layers for which a tensor is generated during the forward pass (e.g., with torch.rand)	
internally_terminated_bool_layers: list of layers for which the output is a single boolean value (used to conditional branching in forward pass)	

layers_computed_with_params: list of layers with trainable parameters

num_tensors_total: number of tensors involved in the forward pass

tensor_fsize_total: total filesize of tensors involved in the forward pass

tensor_fsize_saved: total filesize of tensors saved by the user during the forward pass

num_tensors_saved: number of tensors saved by the user during the forward pass

total_param_tensors: number of tensors consisting of trainable parameters

total_param_layers: number of layers with trainable parameters (can differ from total_param_tensors since a layer can have multiple parameter tensors; e.g., a convolutional layer has both kernel weights and bias terms)

total_params: number of trainable parameters in the model

total_params_fsize: total filesize of trainable parameters in the model

module_addresses: list of modules in the model

module_types: the class of each module in the model

module_num_passes: the number of times each module is called

module_pass_children: for each module, the list of submodules it contains

top_level_module_passes: the list of passes through any module that are not

tensor_shape: dimensions of the tensor returned by this operation

tensor_dtype: datatype of the tensor returned by this operation

tensor_fsize: filesize of the tensor returned by this operation

creation_args: the positional arguments to the function applied for this operation

creation_kwargs: the keyword arguments to the function applied for this operation

func_applied: pointer to the function applied for this operation

func_applied_name: the name of the function applied for this operation

func_time_elapsed: the amount of time elapsed during the function call for this operation

func_rng_states: state of all random number generators during the function call (i.e., so that stochastic functions can be re-run and yield the same outputs)

func_position_args_non_tensor: non-tensor positional arguments to the function applied for this operation

func_keyword_args_non_tensor: non-tensor keyword arguments to the function applied for this operation

function_is_inplace: whether the function modifies its input tensor in-place (i.e., as opposed to creating a new tensor)

gradfunc_name: name of the grad function for this operation

is_part_of_iterable_output: whether the

contained in another module

pass_start_time: timestamp when the forward pass started

pass_end_time: timestamp when the forward pass ended

elapsed_time_total: total elapsed time during the forward pass

elapsed_time_function_calls: total elapsed time for the tensor operations in the model (excluding time that *TorchLens* spent performing logging and post-processing operations)

elapsed_time_torchlens_logging: total time spent by *TorchLens* with logging and post-processing operations

operation returned an iterable (e.g., list or tuple) of tensors instead of a single tensor

iterable_output_index: if the operation returned an iterable output, the index of this output for this operation (e.g., 2 for the third entry, based on 0-based indexing)

computed_with_params: whether the operation involved trainable parameters

num_param_tensors: number of trainable parameter tensors for the operation (e.g., 2 for a convolutional layer with separate weight and bias parameters)

parent_param_shapes: shapes of any parameter tensors involved in the operation

num_params_total: total number of trainable parameters for the operation

parent_params_fsize: total filesize of parameters involved in this operation

same_layer_operations: list of operations corresponding to the same layer as this one (e.g., if the operation is conv2d_3_5:2, and the layer conv2d_3_5 has three passes, the other operations will be conv2d_3_5:1, and conv2d_3_5:3)

parent_layers: layers whose outputs are inputs to this operation

orig_ancestors: ancestors of this operation that have no inputs (e.g., input layers, layers for internally generated tensors)

child_layers: layers that take this layer’s outputs as inputs

sibling_layers: layers sharing a parent

	layer with this operation<br><br>*spouse_layers*: layers sharing a child layer with this operation<br><br>*is_input_layer*: whether the operation is part of an input layer<br><br>*has_input_ancestor*: whether the operation has an input layer ancestor (i.e., whether it is computed from the inputs, as opposed to being computed solely from internally generated tensors)<br><br>*min_distance_from_input*: minimum number of operations separating the operation from an input<br><br>*max_distance_from_input*: maximum number of operations separating the operation from an input<br><br>*is_output_layer*: whether the operation returns the output of the model<br><br>*is_output_ancestor*: whether the operation is the ancestor of an output layer (i.e., as opposed to producing an internally terminated tensor)<br><br>*output_descendents*: output layers descended from this operation<br><br>*min_distance_from_output*: minimum number of operations separating this layer from the output<br><br>*max_distance_from_output*: maximum number of operations separating this layer from the output<br><br>*is_buffer_layer*: whether the operation corresponds to a saved buffer tensor<br><br>*buffer_address*: if a buffer layer, the address of the layer within the model data

	structure<br><br>*initialized_inside_model*: whether the operation initializes a new tensor inside the model with no parent tensors (i.e., using torch.ones or torch.rand)<br><br>*internally_initialized_parents*: any parent layers that are internally initialized<br><br>*internally_initiailized_ancestors*: ancestors of the model that are internally initialized<br><br>*terminated_inside_model*: whether the operation has no children and terminates inside the model<br><br>*is_terminal_bool_layer*: whether the operation both has no children and returns a single boolean value (used by *TorchLens* to infer conditional branching)<br><br>*in_cond_branch*: whether the operation is involved in evaluating a conditional (if-then) operation<br><br>*is_computed_inside_submodule*: whether the operation is performed inside a submodule of the model<br><br>*containing_module_origin*: the module in which the operation was performed, if any<br><br>*containing_module_origin_nested*: the nested set of modules in which the operation was performed<br><br>*modules_entered*: modules entered by the output tensor for this operation<br><br>*is_submodule_output*: whether the output of the operation exits a submodule<br><br>*modules_exited*: modules exited by the output tensor for the operation

	is_bottom_level_submodule_output: whether the operation belongs to a module containing a single tensor operation (e.g., a module containing a single convolution operation)
